# Cognitive function in Nigerian children with newly diagnosed epilepsy: a preliminary report

**DOI:** 10.11604/pamj.2016.24.113.8995

**Published:** 2016-06-02

**Authors:** Ike Oluwa Abiola Lagunju, Yetunde Celia Adeniyi, Gbemi Olukolade

**Affiliations:** 1 Department of Paediatrics, College of Medicine, University of Ibadan, Ibadan, Nigeria & Department of Paediatrics, University College Hospital, Ibadan, Nigeria; 2 Department of Child and Adolescent Psychiatry, University College Hospital, Ibadan, Nigeria

**Keywords:** Epilepsy, cognitive functions, WISC-IV, intellectual disability

## Abstract

**Introduction:**

Epilepsy has long been associated with cognitive dysfunction and educational underachievement. The purpose of the study was to describe the baseline findings from a larger prospective study.

**Methods:**

New cases of epilepsy aged 6-16 years seen at a paediatric neurology clinic in Ibadan, Nigeria were evaluated for any evidence of cognitive impairment. Intelligence quotient (IQ) of the participants was measured using the Wechsler Intelligence Scale for Children-Fourth Edition (WISC-IV). Scores on cognitive subtests and Full Scale IQ (FSIQ) were computed and association between the subsets scores and seizure variables were calculated.

**Results:**

40 children, 24 males and 16 females were studied and their ages ranged from 6 to 16 years with a mean of 10.8 (SD=3.0) years. Global intellectual functioning as measured by the WISC-IV was in the normal range (FSIQ scores <85) for 52.5% (n = 21) of the participants and the remaining participants (47.5%) scored between the borderline and severe category for intellectual disability. The strongest correlation was between ‘caregiver's assessment of school performance’ and FSIQ, (r = 0.70; p< 0.001). Age at onset of epilepsy and seizure type had no significant association with scores on the WISC-IV composite scores.

**Conclusion:**

There is a high prevalence of significant cognitive dysfunction in Nigerian children with epilepsy, even in the absence of any known brain insult. All children with epilepsy should have routine IQ assessment following diagnosis, in order to allow for early intervention when indicated, and thus, improved outcomes.

## Introduction

Epilepsy has long been associated with cognitive dysfunction and academic underachievement [1–5]. Numerous studies carried out among children with epilepsy have demonstrated an association between epilepsy and cognitive deficits [6–8]. A number of factors have been identified as being responsible for the cognitive problems in epilepsy and these factors include type of epilepsy, underlying pathology that predisposed the brain to epilepsy, age at onset of epilepsy, frequency of seizures, duration of epilepsy and anti-epileptic drugs (AEDs)use [4, 9–11]. Younger age at onset of seizures has been reported to be one of the most important predictors of cognitive dysfunction in children with epilepsy [2, 10, 12]. There is no consensus on what age should be considered early, but studies have reported that children whose seizures started before one year of age are at a significantly higher risk of cognitive problems as a result of the vulnerability of the infant brain to insult [13, 14]. Other social and environmental factors have been reported to contribute to the academic underachievement and overall poor school performance seen in children and adolescents with epilepsy. These factors include stigma, low self-esteem and school attendance difficulties as a result of the seizure episodes and medical appointments [15–20]. Tests of intelligence like the Wechsler Intelligence Scales for Children give a global measure of intellectual abilities and have been widely used among children and adolescents with epilepsy [4, 21–23]. These scales also assess specific areas of cognitive function such as processing speed, working memory, verbal and performance tasks. Apart from providing information on the different cognitive domains, the scales are useful in providing insight into the academic problems of children with epilepsy and can also serve as baseline for future comparison [4]. The aim of this study was to describe cognitive functions of children and adolescents with epilepsy attending a paediatric neurology clinic in a resource-poor country and to find the associations between cognitive functions and seizure variables. This is a preliminary report of our initial findings in a prospective longitudinal study.

## Methods

### Participants

Children aged between 6 and 16 years newly diagnosed with epilepsy were recruited using consecutive sampling method from the outpatient clinic of the paediatric neurology unit, University College Hospital, Ibadan, Nigeria. Children with epilepsy who had a history suggestive of underlying brain insult and thus classified as remote symptomatic epilepsy were excluded from the study. The paediatric neurologist evaluated all the cases and diagnosis of epilepsy was based on an accurate eyewitness account and the electroencephalographic (EEG) findings. Classification of epilepsy type was based on the International League Against Epilepsy criteria [24]. School and seizure-related information were taken from the participants’ caregivers. Informed consent was obtained from caregivers before enrolment into the study.

### Measure of intelligence quotient

Intelligence quotient of the participants was measured using the Wechsler Intelligence Scale for Children-Fourth Edition (WISC-IV). The Wechsler Intelligence Scale for Children (WISC), developed by David Wechsler, is an intelligence test for children between the ages of 6 and 16 years [25]. The WISC-IV generates a full IQ score from four index scores. It has four main components that are referred to as Indexes. These are the Verbal Comprehension Index, the Perceptual Reasoning Index, the Working Memory Index and the Processing Speed Index. Within each of these four domains are a variety of sub-tests that add up to form the index score. Each participant was administered the 10 core subtests of the WISC-IV, a raw score was generated by summing all the correct items from each subtest. The raw scores were converted into scaled scores, the scaled scores for each Index were then converted into IQ scores which can be directly comparable across age groups. The child psychiatrist (YA) and the clinical psychologist (GO) performed IQ assessments in all the cases. Both had been trained and are proficient on the use of WISC-IV. Feedback was provided to each family based on the outcome of the assessment and recommendations were made to the parents regarding academic improvements. The study conformed to the World Medical Association declaration of Helsinki on Ethical principle for medical research. Ethical approval was given by the UI/UCH Ethics Research Committee.

### Statistical analysis

Analysis was done using the Statistical Package for the Social Sciences (SPSS, Chicago, IL, USA - Version 20.0). Frequencies, means and standard deviation were used to summarize school-related, seizure-related data and WISC-IV composite scores. Association between intellectual functioning (WISC-IV composite scores) and school-related and seizure-related variables were analysed using the two-tailed Pearson's correlation test/two-tailed Spearman correlation tests for continuous data (age at first seizure, duration of seizure before presentation) and independent t-test/ANOVA for categorical variables (seizure type, appropriate class for age, caregiver's assessment of school performance, having repeated a class in the past). Level of significance was set at p < 0.05.

### Limitations

The current report was derived from a cross-sectional evaluation and represents a preliminary report of a prospective study. Consequently, causal inferences cannot be made and it will be more informative to assess these participants at different periods in the course of management and the findings compare with these baseline findings. In addition, the small sample size does not allow for generalising our findings to the population of Nigerian children with epilepsy.

## Results

Forty children, 24 males and 16 females, who were newly diagnosed with epilepsy were recruited into the study and their ages ranged from 6 to 16 years with a mean of 11.8 years (SD=3.0 years). The main school-related characteristics (class appropriateness for age, having repeated a class, caregiver satisfaction with performance) and seizure-related characteristics (age at first seizure, seizure type, interval between first seizure and presentation) are presented in Table 1. Eighteen (45%) of the caregivers expressed dissatisfaction with participants’ school performance and 19 (47.5%) classified the performance of their wards as either poor (17.5%) or below average (30%). Eight (20%) of participants were reported to have repeated a class at one time or the other and 2 (5%) were yet to be enrolled in school because of fears about stigma.

**Table 1:** Demographic and school characteristics in 40 children with epilepsy

Characteristic	Number of cases	%
*Gender*		
Male	24	60.0
Female	16	40.0
*Is child's class appropriate for age*		
Yes	27	67.5
No	11	27.5
Not enrolled in school	2	5.0
*Caregivers’ assessment of school performance*		
Very good	7	17.5
Above average	6	15.0
Average	6	15.0
Below average	12	30.0
Poor	7	17.5
Not enrolled in school	2	5.0
*Has child repeated a class?*		
Yes	8	20.0
No	30	75.0
Not in school	2	5.0

### Seizure-related characteristics

Seizure-related characteristics of the participants are shown in Table 2. The mean age at onset of epilepsy was 80.83 (SD = 4.80) months and the interval between onset of afebrile seizures and presentation at our facility ranged from 1 to 137 months with a median of the 11.5 months. Twenty two (55%) had partial epilepsy, with secondarily generalized seizures in 14 (35%) of them. The remaining 18 (45%) patients had generalized epilepsy.

**Table 2:** Seizure variables in 40 children with newly-diagnosed epilepsy

Seizure characteristics	Number	%
*Age at first a febrile seizure*		
< 1year	3	7.5
1-5 years	10	25.0
>5 years	27	67.5
*Epilepsy type*		
***Generalised***		
GTC	17	42.5
Atonic	1	2.5
***Partial***		
Partial with secondary generalisation	14	35.0
Complex partial	5	12.5
Simple partial	3	7.5

### WISC IV composite scores results

**Figure 1:**
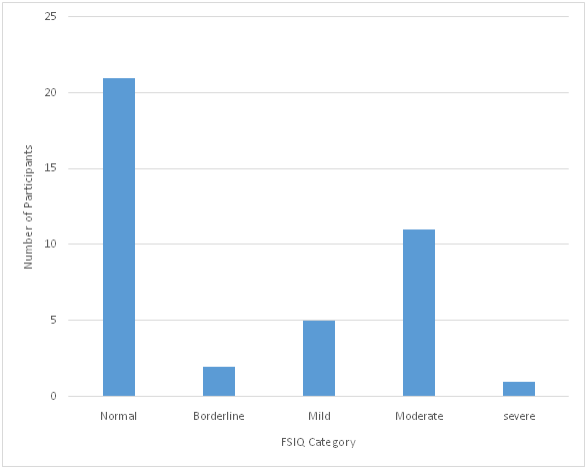
The distribution of Full Scale IQ among the participants

Global intellectual functioning as measured by the WISC-IV was normal (FSIQ scores >84) in 21 (52.5%) of the children. Two(5.0%) had scores in the borderline intellectual disability (ID) category (FSIQ scores between 70 and 84) and 5 (12.5%) in the mild ID category (FSIQ scores between 50 and 69), 11 (27.5%) in the moderate ID category. Only 1 (2.5%) of the participants had a FSIQ in the severe category (Figure 1). Table 3 shows the composite and subtest scores for the participants. The mean FSIQ score for the participants was 71.48 (SD = 22.75). The highest composite scores on the different scales were in both the Perceptual Reasoning Index (PRI) and Verbal Comprehension Index (VCI) scales with mean scores of 15.90 (SD = 9.69) and 17.02 (SD = 9.98) respectively. The lowest mean score (7.62; SD = 4.17) was recorded in the Working Memory Index (WMI). Means were also calculated for each of the ten sub-tests and results are shown in Table 3. Highest mean scores were recorded in Picture Concept (7.20; SD = 4.65) and Matrix Reasoning (7.20; SD = 4.60) subtests. Participants performed lowest in Block Design and Digit Span with mean scores of 3.35 (SD = 2.12) and 3.93 (SD = 2.19) respectively.

**Table 3:** WISC IV Composite and subtests mean scores of the participants

WISC Scales and subtests	N=40 Mean (SD)
Composite scores	
Verbal Comprehension IQ	15.90 (9.69)
Perceptual Reasoning IQ	17.02 (9.98)
Working Memory IQ	7.62 (4.17)
Processing Speed IQ	10.15 (6.50)
Full Scale IQ	71.48 (22.75)
Verbal Comprehension Subtests	
Similarities	5.55 (3.14)
Vocabulary	5.60 (3.80)
Comprehension	5.63 (3.58)
Perceptual Reasoning Index	
Block Design	3.35 (2.12)
Picture Concept	7.20 (4.65)
Matrix Reasoning	7.20 (4.60)
Working Memory Index	
Digit Span	3.93 (2.19)
Letter-Number Sequencing	4.03 (2.60)
Processing Speed Index	
Coding	5.35 (3.79)
Symbol Search	5.40 (3.19)

### Results related to school and seizure variables

Table 4 and Table 5 shows the results of the independent t test and one-way ANOVA across the different variables. Caregivers were asked to arbitrarily categorize the participants’ performance in school to ‘poor’, ‘below average’, ‘average’, ‘above average’ and ‘very good’. The mean scores were highest for participants that were rated very good (between 12.14 and 89.29) and lowest for those rated ‘poor’ (between 4.14 and 55.14) on all the domains of the WISC-IV. Using a one-way ANOVA, the difference in means was found to be statistically significant across all the WISC-IV domains. There was also a statistically significant difference in the mean scores of participants based on the caregivers’ report of their satisfaction with school performance, the significance was also found across all the domains; VCI (t= 4.524; p < 0.001), PRI (t= 3.887; p < 0.001), WMI (t=3.806; p =0.001), PSI (t= 4.080; p < 0.001). No significant difference was found in the mean scores in the different seizure types and whether participants’ current class was appropriate for age or not. Using the Pearson's correlation coefficient, no significant association was found between age at first seizure or duration of seizure before presentation and the WISC-IV composite scores.

**Table 4:** WISC-IV scores versus seizure type and school-related variables

Variables	Seizure Type			Is Child class appropriate for age?			Has child repeated a class?		
	Generalised	Partial		Yes	No		Yes	No	
Scales	Mean (SD)	Mean (SD)	t (p value)	Mean (SD)	Mean (SD)	t (p value)	Mean (SD)	Mean (SD)	t (p value)
VCI	17.83 (8.96)	14.29 (10.43)	1.129 (0.266)	18.25 (9.32)	12.27 (5.09)	1.808(0.079)	5.88 (2.17)	19.36 (8.67)	4.324(<0.001)
PRI	18.89 (7.90)	15.42 (14.63)	1.068 (0.292)	19.56 (9.80)	12.91 (8.69)	1.955(0.058)	4.57 (1.59)	20.13 (9.40)	3.445(0.001)
WMI	8.33 (3.51)	7.24 (4.66)	0.817 (0.419)	8.59 (4.13)	6.18 (3.66)	1.685(0.101)	4.50 (2.62)	8.80 (3.97)	2.886 (0.007)
PSI	11.06 (5.42)	9.67 (7.39)	0.659 (0.514)	11.78 (6.65)	7.20 (5.10)	2.013(0.052)	4.38 (2.62)	12.10 (6.36)	3.376(0.001)
FSIQ	76.33 (20.82)	66.38 (23.95)	1.373(0.178)	80.41 (19.20)	55.09 (19.01)	3694(0.001)	53.63 (22.10)	78.27 (19.45)	3.097(0.004)

VCI: Verbal Comprehension Index; PRI: Perceptual Reasoning Index; WMI: Working Memory Index; PSI: Processing Speed Index; FSIQ: Full Scale Intelligence Quotien

**Table 5:** WISC-IV scores versus caregivers’ satisfaction with, and assessment of school performance

Variable	* Caregiver satisfied with school performance?		** Caregiver's assessment of school performance						
	*Yes*	*No*		*Poor*	*Below Average*	*Average*	*Above average*	*Very Good*	
Scale VCI	*Mean (SD)*	*Mean (SD)*	*t (p value)*	*Mean (SD)*	*Mean (SD)*	*Mean (SD)*	*Mean (SD)*	*Mean (SD)*	*F (p value)*
	21.90 (7.62)	10.56 (7.83)	4.524 (<0.001)	7.57 (6.45)	15.17 (9.39)	18.33 (8.80)	20.33 (9.13)	22.57 (7.85)	3.344 (0.021)
PRI	22.65 (8.88)	12.06 (7.80)	3.887(<0.001)	8.29 (4.92)	15.50 (8.39)	18.33 (9.46)	20.50 (8.60)	27.57 (8.66)	5.318 (0.002)
WMI	9.95(3.52)	5.61 (3.50)	3.806(0.001)	4.14 (2.27)	6.33 (3.28)	9.17 (53)	9.19 (3.06)	12.14 (2.85)	6.848 (0.001)
PSI	13.90(6.02)	6.67 (4.75)	4.080(<0.001)	5.29 (2.63)	8.17 (5.44)	12.33 (6.31)	12.33 (7.111)	16.43 (5.86)	4.360 (0.006)
FSIQ	83.85(4.94)	61.11 (23.12)	3.637(0.001)	55.14 (22.06)	67.92 (22.39)	80.33 (19.33)	87.50 (7.91)	89.29 (22.38)	2.790 (0.012)

* Independent t test

** Analysis of Variance

VCI: Verbal Comprehension Index; PRI: Perceptual Reasoning Index; WMI: Working Memory Index; PSI: Processing Speed Index; FSIQ: Full Scale Intelligence Quotient

## Discussion

This study is part of a larger prospective, longitudinal study with an overall aim of investigating changes in the cognitive functions of children and adolescent with epilepsy. This current paper is a preliminary report describing our baseline findings. The study showed that nearly half of the participants had abnormal scores on the test of intelligence. This is lower than the 62% reported by Guzeva and colleagues in a cohort of Russian children with epilepsy [26]. Their study however included a larger proportion of children with symptomatic epilepsy and this probably accounts for the very high prevalence of cognitive impairment reported in the cohort. On the other hand, it has been reported that the cognitive function of children with idiopathic generalised epilepsy is usually in the normal range but tends to be somewhat lower than the general population, [27] Our study showed cognitive impairment in nearly half of our patients even in the absence of any identifiable underlying brain injury. One possible reason for this could be because the study site is a referral centre and patients in the high-risk category (those with poorly-controlled seizures and impaired academic functioning) are likely to constitute a significant number of patients seen in our clinic. Another explanation for the poor performance might be due to the inherent cognitive problems that have been associated with epilepsy. It has been reported that children with epilepsy tend to perform poorly on tests of intelligence, experience significant academic under achievement and have a long-term risk of learning difficulties [1–5, 28]. The study showed no significant association between age of onset of epilepsy and performance on IQ test. Many studies have attempted to link age of onset of epilepsy to cognitive functioning and academic underachievement, with conflicting and inconclusive findings; while some studies have found no significant associations between age of onset of epilepsy and cognitive functions [2, 10, 12], others have found significant association, especially if the epilepsy starts before the age of 3 years [4, 29, 30]. The mean age of onset of seizures of 6.7 years in our study is considerably higher than the 3 years reported in the other studies with significant associations [29, 30]. This might explain the absence of any significant association between the age of onset of epilepsy and performance on intellectual test in this study.

Our study showed that the children in the cohort scored higher in the Perceptual Reasoning, Verbal Comprehension Indexes than the Processing speed and Working memory indexes. Our observation is consistent with previous reports [4, 31]. In a recent study, Lopes et al [4] compared 30 children with frontal lobe epilepsy (FLE) with normal children and found that the children in the FLE group scored significantly lower on the processing speed and full scale indexes of the WISC. Processing speed and working memory are seen as an important part of mental ability known as Cognitive Proficiency and children with epilepsy tend to perform poorly on these indexes [31]. One of the strongest associations found in this study was between the composite scores of the WISC-IV and caregiver's arbitrary rating of school performance. This observation is important because it suggests that the academic performance of the patients can be monitored using the caregivers’ assessment. Children get an opportunity for standardized assessment about once a year in our center due to inadequate personnel and cost of the test. Our findings therefore suggest that the caregivers’ assessment can be used routinely as a rough guide to monitor child's progress so that decline can be identified early in a setting of limited resources as seen in Africa.

## Conclusion

Significant cognitive dysfunction occurs frequently in Nigerian children with epilepsy even in the absence of any identifiable underlying insult to the brain. Caregivers’ assessment of child's performance and child having repeated a class showed significant correlations with the IQ rating. Our findings suggest the need for routine IQ assessment in all children diagnosed with epilepsy in order to promote early diagnosis and prompt intervention for improved outcomes. In the setting of limited resources, children with epilepsy whose caregivers’ express dissatisfaction with their academic performance should be given a high priority of IQ assessment.

### What is known about this topic

- Epilepsy is associated with impaired cognitive function;
- Age at onset of epilepsy, anti-epileptic drug therapy and presence of underlying brain injury have been reported to contribute to the impaired cognitive function in epilepsy;
- Social and environmental factors play a role in the cognitive dysfunction seen in children with epilepsy.

### What this study adds

- Impaired intelligence quotient is seen in nearly half of Nigerian children with epilepsy, even in the absence of any identifiable prior brain insult;
- There is a strong correlation between caregiver's perception of academic performance and the performance on the IQ tests;
- In the setting of limited resources, children with epilepsy whose caregivers’ express dissatisfaction with their academic performance should be given a high priority of IQ assessment.
